# 

**Published:** 1870-06

**Authors:** 


*CONTENTS:*

Original Communications:

Art. I. Colloid Tumor of the Broad Ligament, etc., under care of John E. Owens, M. D........ 321
" II. Tubular Pregnancy. By W. C. Hunt, M. D............... 328
" III. Poisoning by Belladonna. By Chas. E. Hogeboom, M. D... 330
" IV. Biliary Calculi.................. 331

Original Translations:

Art. I. Legros and Onimus upon the Influence of Electrical Currents upon the Nervous System. Continued from May Number, 1870............. 331

Foreign Correspondence:

Letter from A. W. Bosworth, Paris... 341

Editor's Book Table................. 344

Editorial: 348

Prof. Tardieu. Ancient Norse Surgery. The Red Blood Corpuscle. Sir James Y. Simpson. Make Haste Slowly. Graduates of Medical Colleges for 1870.

Convention Proceedings.

Convention of Medical Teachers, held in Washington, D.C., April 29, 1870.. 353

Illinois State Medical Society, held at Dixon, Ill., May 17, 1870............. 356

Indiana Medical Society, held at Indianapolis, May 18, 1870................ 367

American Medical Association, Twenty-first Annual Convention, held at Washington, D.C., May 3, 1870....... 367

